# A Machine Learning Approach to Predictive Modelling of Student Performance

**DOI:** 10.12688/f1000research.73180.2

**Published:** 2022-05-23

**Authors:** Hu Ng, Azmin Alias bin Mohd Azha, Timothy Tzen Vun Yap, Vik Tor Goh

**Affiliations:** 1 Faculty of Computer and Informatics, Multimedia University, Cyberjaya, Selangor, 63100, Malaysia; 2 Faculty of Engineering, Multimedia University, Cyberjaya, Selangor, 63100, Malaysia

**Keywords:** Student performance, data mining, support vector machine, naïve bayes, multilayer perceptron

## Abstract

**Background** - Many factors affect student performance such as the individual’s background, habits, absenteeism and social activities. Using these factors, corrective actions can be determined to improve their performance. This study looks into the effects of these factors in predicting student performance from a data mining approach. This study presents a data mining approach in identify significant factors and predict student performance, based on two datasets collected from two secondary schools in Portugal.

**Methods** – In this study, two datasets  are augmented to increase the sample size by merging them.  Following that, data pre-processing is performed and the features are normalized with linear scaling to avoid bias on heavy weighted attributes.  The selected features are then assigned into four groups comprising of student background, lifestyle, history of grades and all features. Next, Boruta feature selection is performed to remove irrelevant features. Finally, the classification models of Support Vector Machine (SVM) , Naïve Bayes (NB) , and Multilayer Perceptron (MLP)  origins are designed and their performances evaluated.

**Results** - The models were trained and evaluated on an integrated dataset comprising 1044 student records with 33 features, after feature selection. The classification was performed with SVM, NB and MLP with 60-40 and 50-50 train-test splits and 10-fold cross validation. GridSearchCV was applied to perform hyperparameter tuning. The performance metrics were accuracy, precision, recall and F1-Score. SVM obtained the highest accuracy with scores of 77%, 80%, 91% and 90% on background, lifestyle, history of grades and all features respectively in 50-50 train-test splits for binary levels classification . SVM also obtained highest accuracy for five levels  classification  with 39%, 38%, 73% and 71% for the four categories respectively. The results show that the history of grades form significant influence on the student performance.

## Introduction

The definition of a student is a person who attends school or any education institution level to achieve a certain level of knowledge or skill set in a course under the supervision of an educator. Almost everyone was once a student with responsibilities to acquire proper education. Acquiring knowledge by means of getting the right education is of utmost importance and each person should have basic equality in receiving education.

When discussing education at the secondary level, a vital aspect to consider is student performance. Student performance can be assessed in a variety of dimensions, either through exam-based assessment or participation-based assessment. Exam-based assessment includes quizzes, midterms, and final exams, while participation-based assessment is a two-way communication during learning and group activities.

Apart from the obvious, there are so many factors that can affect student performance, such as individual habits, absenteeism, social activities after school and others. This gives way to having machines to learn patterns from data so that they can predict how well a student performs; by acknowledging these factors and subsequently detecting and improve their performance as early as possible.

The contributions of this paper are the identification of significant features that influence student assessment, which in turn can be used to develop various predictive models to ascertain student performance. This will assist educators to form corrective or remedial actions can help to improve student performance. In addition, this may also assist in formulating curriculums that may direct students to career pathways that are most suitable for them.

The paper is structured as follows: Related Works, Methodology and Results, followed by Conclusions.

### Related Works

Student performance is an essential part in a secondary-level education as it will show where the student stands when continuing to higher education. Daud *et al.* ^1^ noted that the ability to predict the success of a student is essential and seems to be a fascinating area to dig into.

Sokkhey *et al.* ^2^ found out that mathematics is one of the subjects that has scientific progression on students. All aspects of human life at various levels are influenced by mathematics and there are no instances in life where mathematics is not used.

Akhtar *et al*. ^3^ discovered that social status is correlated with family’s social and monetary wealth. They managed to find the effect of monetary wealth on students’ grade in Pakistan.

Amazona *et al.* ^4^ as well as Hussain *et al.* ^5^ have adopted educational data mining (EDM) methods to perform gathering, achieving, and studying of information concerning student’s assessment and learning.

On another note, researchers have also looked at student dropout, ^6^ interpersonal influences ^7^ as well as career decisions after graduation albeit at the tertiary level. ^8^^–^^10^

Exploratory data analysis (EDA) ^11^ is a method of analyzing dataset to summarize the important features *via* visualization. EDA helps:

• to find errors.
• to check assumptions.
• to determine the tentative choice of suitable models and tools.
• to determine the relationship between the dependent and independent variables.
• to detect the directions and size of the relationship between variables.

Feature selection is a component of dimensionality reduction where it reduces the number of features to maximize the performance of a machine learning model. Too many features in a dataset can overwhelm a machine learning classifier and potentially reduce the efficacy. ^12^

The Boruta feature algorithm is a wrapper algorithm that underpins the random forest model. From the results yielded by Tang *et al.,* ^12^ feature selection is able to effectively recognize and improve overall evaluation metrics on their medical dataset research.

Support Vector Machine (SVM) is able to build the best possible boundary of a line called hyperplanes, which can segregate dimensional spaces into classes. In the work of Sekeroglu *et al*., ^13^ they achieved good results with SVM on Mathematics and Portuguese subjects from two secondary schools.

Naïve Bayes (NB) is based on Bayes rule of conditional probability and has high capabilities in dealing big datasets. ^4^ The method is used to estimate the probability of a property given set of data as proof and Bayes’ theorem. The posterior is calculated from the product of likelihood and prior and divisible by its evidence.

Multilayer perceptron (MLP) underpins the artificial neutral network (ANN). ^4^ It has an interconnection of perceptron in which it flows from the input to the output in a single direction with multiple routes.

### Methodology and Results

**Figure 1.**
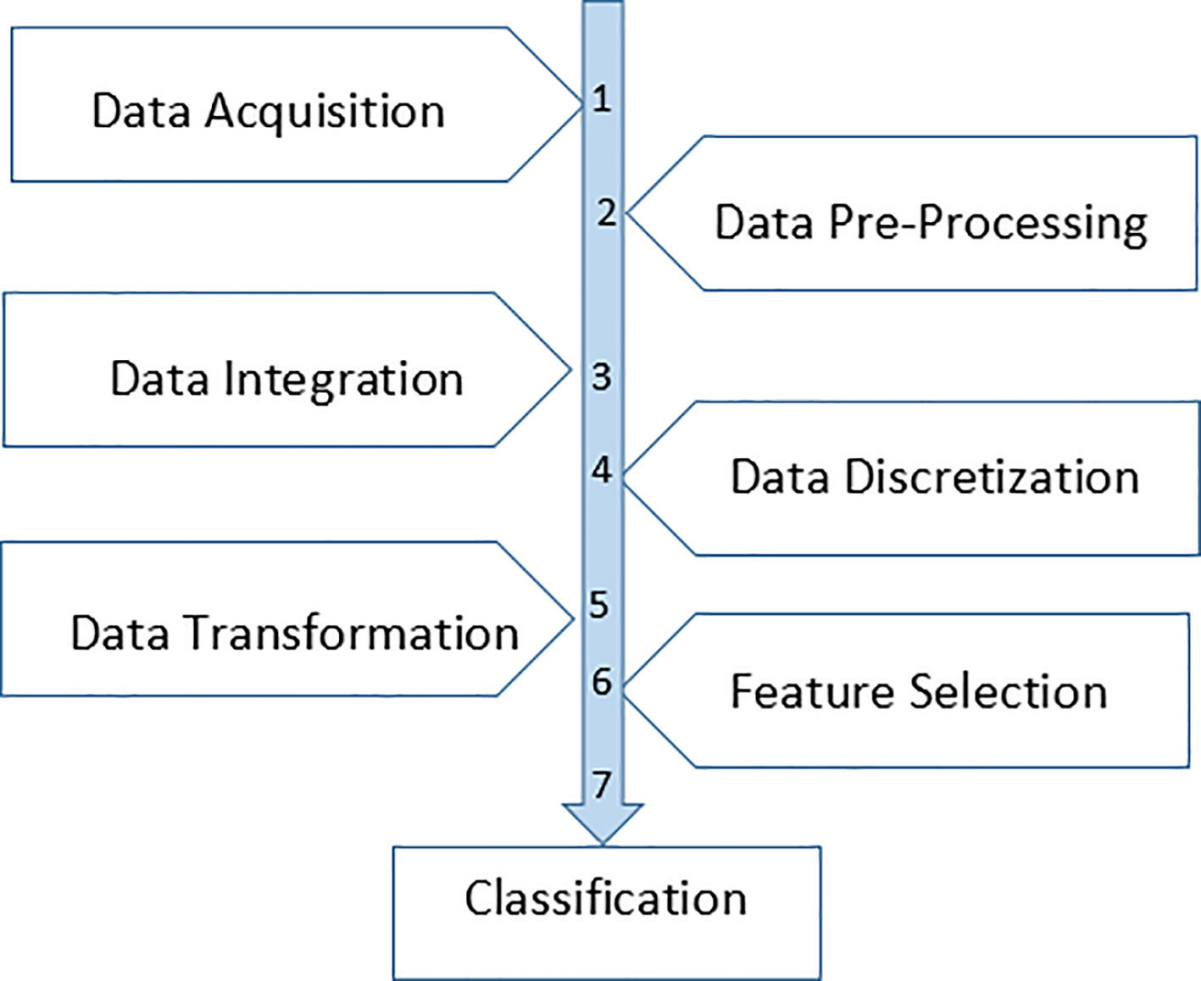
Flow of the processes.

In this research work, the approach consists of seven stages, namely data acquisition, data processing, data integration, data discretization, data transformation, feature selection and classification. The flow of the research is shown in Figure 1.

a) Data acquisition

**Figure 2.**
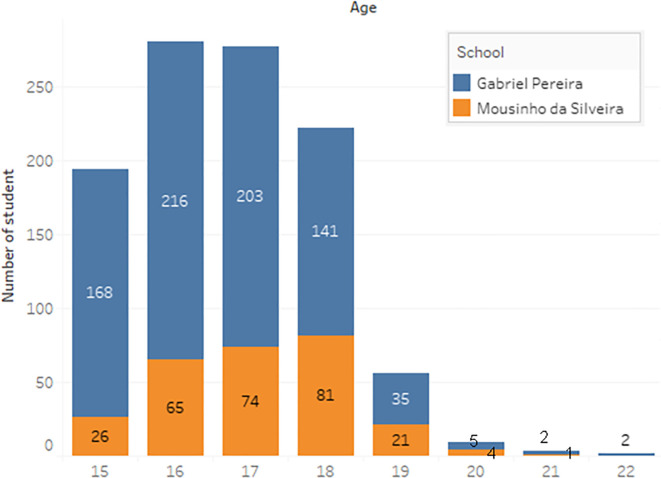
Distribution of age.

**Figure 3.**
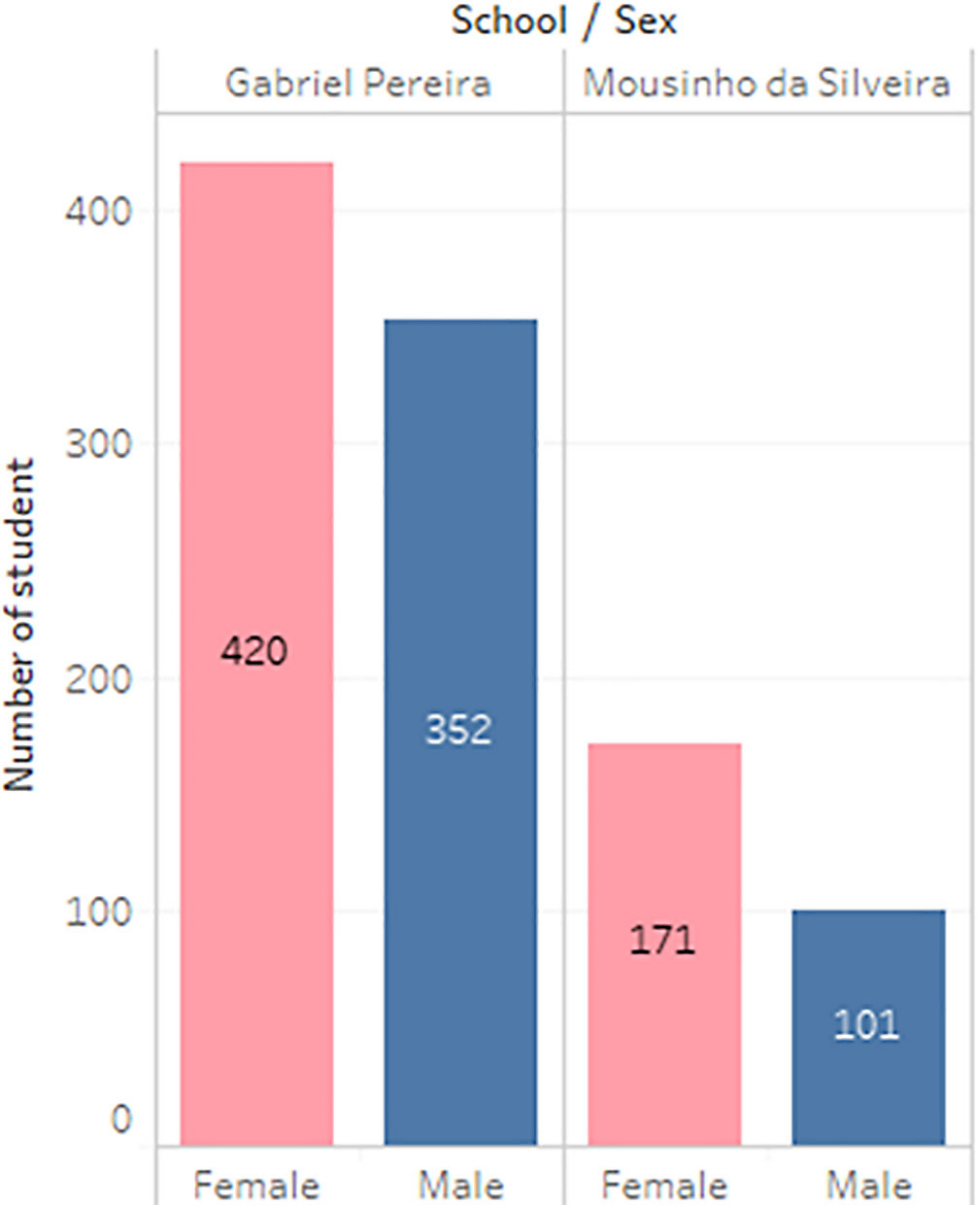
Distribution of gender.

**Figure 4.**
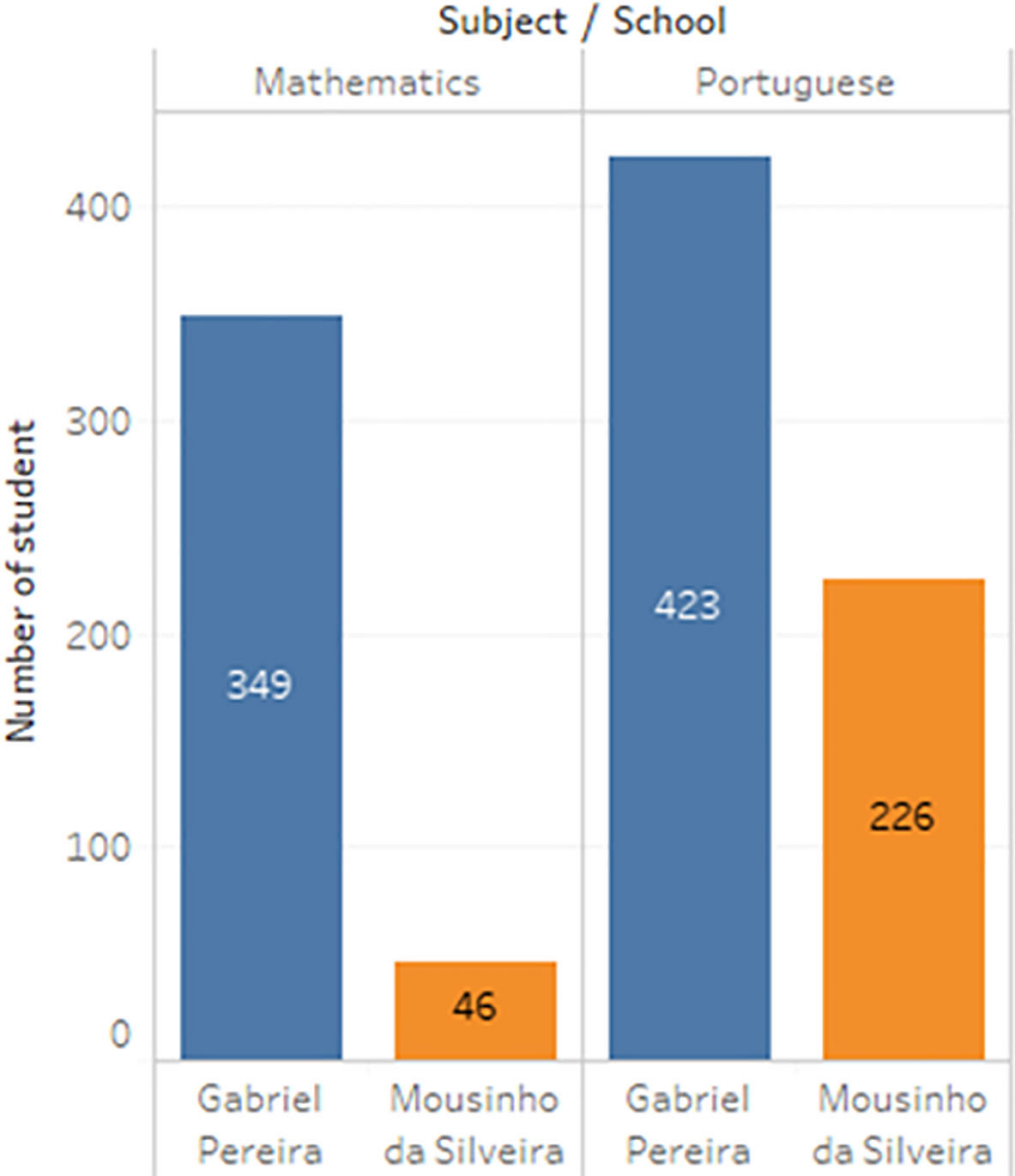
Distribution of subject.

**Figure 5.**
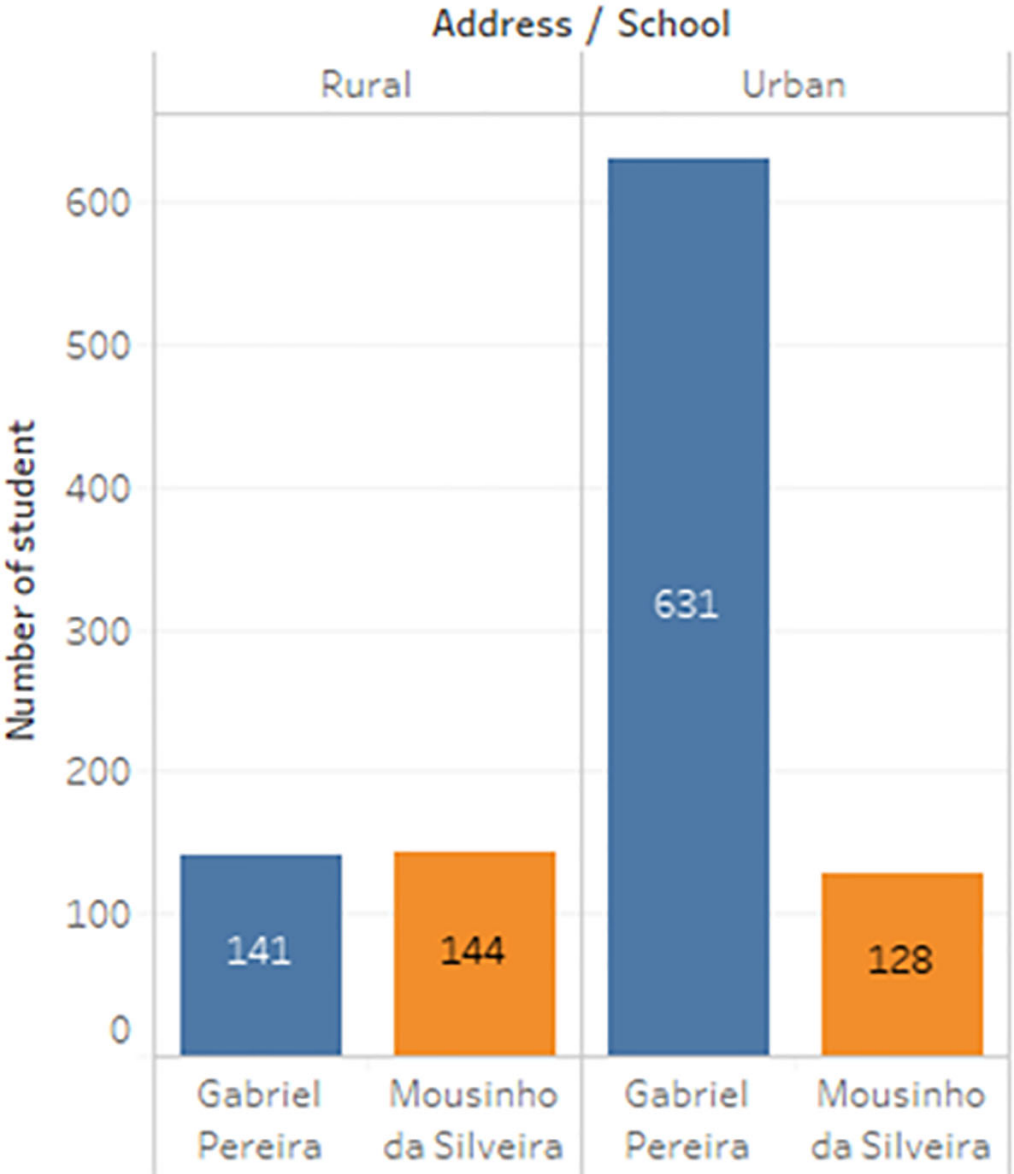
Distribution of student accommodation.

**Figure 6.**
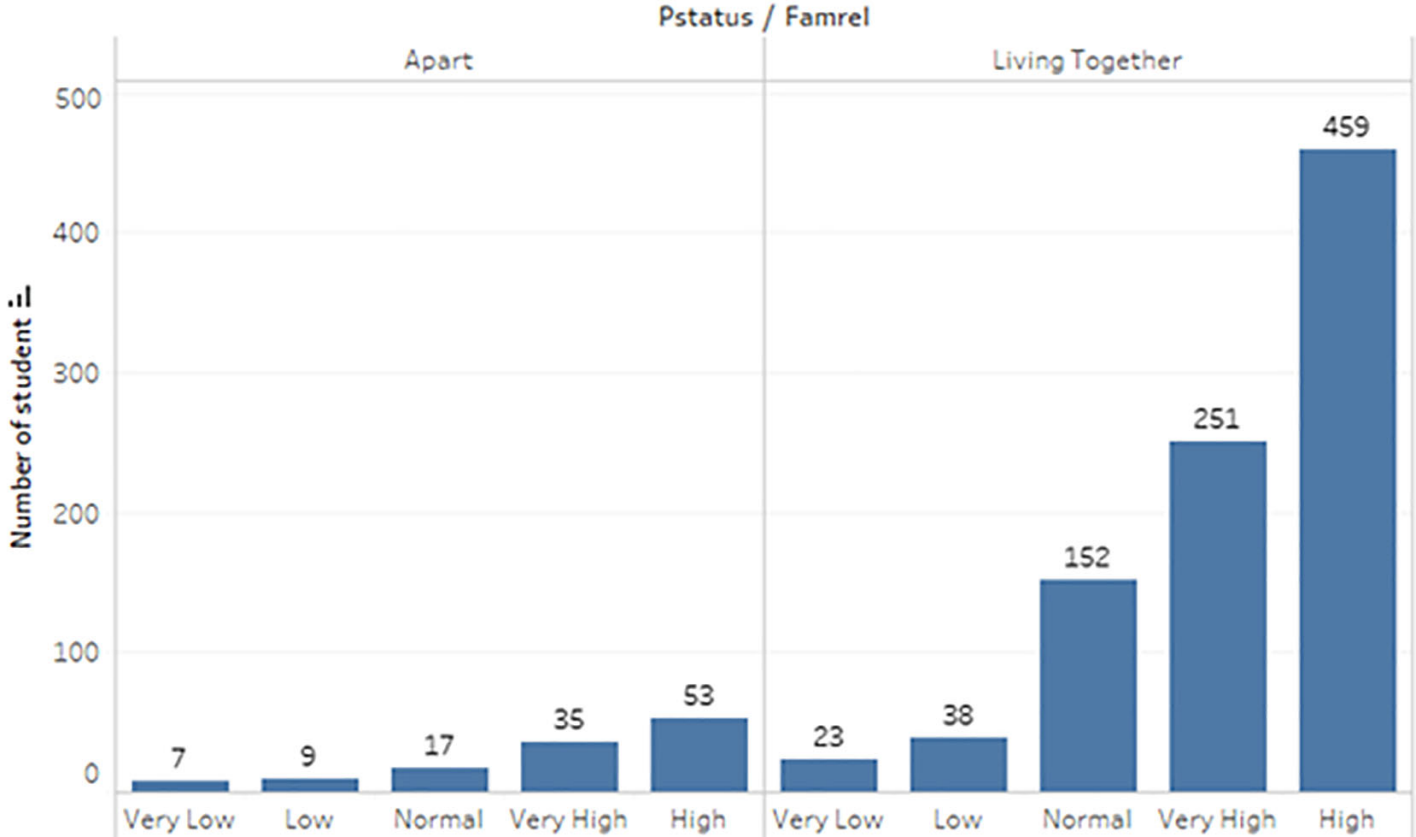
Distribution of relationship with parents.

The dataset of student performance is taken from a population of two Portuguese secondary schools namely Gabriel Pereira Secondary School (395 students) ^14^ and Mousinho da Silveira Secondary School (649 students). ^15^ In the survey, the students were taking the subjects, Mathematics and Portuguese. The two datasets were combined and consisted of 1044 students’ personal data and scores for the two subjects. The datasets are visualizations and shown in Figures 2 to 6.

b) Data processing

This process helps to validate the two datasets by making sure there is no missing term in any feature.

c) Data integration

The two datasets were combined and consisted of 1044 students’ records with 33 features. By adopting EDA, ^11^ the selected features are then assigned into four groups comprising of student background (12 features), lifestyle (18 features), history of grades (three features) and all features. Tables 1 to 3 shown the features in student background, lifestyle, history of grades respectively. The category ‘all’ consists of the entire 33 features.

**Table 1.** Student background.

Feature	Description	Value
sex	Gender of student	Male or Female
age	Age of student	15–22
school	School of student	Gabriel Pereira or Mousinho da Silveira
address	Type of student’s home address	Urban or Rural
famsize	Size of family	≤3 or >3
Pstatus	Parent’s cohabitation status	Living together or apart
Medu	Education of parents	None, Primary education, 5 ^th^ to 9 ^th^ grade, Secondary education, Higher education
Fedu		
Mjob	Job of parents	At home, Civil services, Teacher, Healthcare related, Other
Fjob		
reason	Reason to choose the school	Close to home, School reputation, Course preference, Other
guardian	Guardian of student	Father or mother, Other

**Table 2.** Student lifestyle.

Feature	Description	Value
traveltime	Travel time from home to school	<15 minutes 15 to 30 minutes 30 minutes to 1 hour >1 hour
studytime	Weekly study time	<2 hours 2 to 5 hours 5 to 10 hours >10 hours
failures	Number of past class failures	n if 1 ≤ n < 3, else 4
schoolsup	Extra educational school support	Yes or no
famsup	Educational support from family	
paid	Extra paid classes within the course subject	
activites	Extra-curricular activities	
nursery	Attended nursery school	
higher	Plans for higher education	
internet	Have internet access at home	
romantic	In a romantic relationship	
famrel	Quality relationship with family	Very low (1) to very high (5)
freetime	Free time after school	
goout	Going out with friends	
Dalc	Weekday alcohol consumption	
Walc	Weekend alcohol consumption	
health	Current health status	
absences	Number of school absences	0–93

**Table 3.** Student history of grades.

Feature	Description	Value
G1	First period grade	0–20
G2	Second period grade	
G3	Final grade	

d) Data discretization

Tables 4 and 5 show the binary levels and 5 levels ^5^ after discretization, representing the grades of the students.

**Table 4.** Binary levels classification.

Ordinal categorical	Value
Pass	10–20
Fail	0–9

**Table 5.** 5 Levels classification.

Ordinal categorical	Value
A	15–20
B	13–14
C	10–12
D	8–9
F	0–7

e) Data transformation

The features are normalized with linear scaling to avoid bias on heavy weighted attributes.

f) Feature selection

Next, Boruta feature selection was performed to remove irrelevant features.

g) Classification

Three supervised machine learning techniques were implemented which are support vector machine, naïve Bayes, and multilayer perceptron 60–40 and 50–50 train-test splits and 10-fold cross validation. Four categories that comprise of student background, student lifestyle, student history of grades (history) and all features. Experiments are carried out on binary levels and five level classification. Binary levels classification will indicate fail or pass, meanwhile for the five levels classification is for student scores F, D, C, B and A.

GridSearchCV is applied to perform hyperparameter tuning. The performance metrics are accuracy, precision, recall and F1-Score. The experiments results are shown from Tables 6 to 11.

**Table 6.** SVM (Binary levels).

Metrics	Background	Lifestyle	History	All
**60 Train – 40 Test**				
Accuracy	0.768	0.789	0.899	0.895
Precision	0.768	0.804	0.928	0.934
Recall	1.000	0.958	0.941	0.929
F1 Score	0.869	0.874	0.934	0.931
**50 Train – 50 Test**				
Accuracy	0.772	0.798	0.908	0.900
Precision	0.772	0.809	0.932	0.931
Recall	0.999	0.966	0.948	0.941
F1 Score	0.871	0.880	0.940	0.936

**Table 7.** SVM (5 Levels).

Metrics	Background	Lifestyle	History	All
**60 Train – 40 Test**				
Accuracy	0.394	0.388	0.742	0.716
Precision	0.195	0.322	0.750	0.715
Recall	0.394	0.388	0.742	0.716
F1 Score	0.246	0.286	0.742	0.708
**50 Train – 50 Test**				
Accuracy	0.389	0.381	0.729	0.708
Precision	0.191	0.329	0.735	0.708
Recall	0.389	0.381	0.729	0.708
F1 Score	0.230	0.300	0.711	0.699

**Table 8.** NB (Binary levels).

Metrics	Background	Lifestyle	History	All
**60 Train – 40 Test**				
Accuracy	0.760	0.785	0.903	0.891
Precision	0.775	0.826	0.954	0.948
Recall	0.968	0.913	0.918	0.907
F1 Score	0.861	0.867	0.935	0.927
**50 Train – 50 Test**				
Accuracy	0.761	0.787	0.907	0.894
Precision	0.779	0.830	0.958	0.948
Recall	0.964	0.911	0.922	0.912
F1 Score	0.861	0.868	0.939	0.930

**Table 9.** NB (5 Levels).

Metrics	Background	Lifestyle	History	All
**60 Train – 40 Test**				
Accuracy	0.373	0.250	0.748	0.515
Precision	0.283	0.263	0.752	0.515
Recall	0.373	0.250	0.748	0.515
F1 Score	0.306	0.166	0.747	0.497
**50 Train – 50 Test**				
Accuracy	0.370	0.257	0.719	0.542
Precision	0.284	0.261	0.747	0.537
Recall	0.370	0.257	0.739	0.542
F1 Score	0.310	0.173	0.740	0.523

**Table 10.** MLP (Binary levels).

Metrics	Background	Lifestyle	History	All
**60 Train – 40 Test**				
Accuracy	0.767	0.787	0.899	0.886
Precision	0.769	0.803	0.928	0.921
Recall	0.995	0.957	0.941	0.934
F1 Score	0.868	0.873	0.934	0.927
**50 Train – 50 Test**				
Accuracy	0.767	0.785	0.906	0.886
Precision	0.773	0.791	0.932	0.919
Recall	0.987	0.982	0.948	0.936
F1 Score	0.867	0.786	0.940	0.927

**Table 11.** MLP (5 Levels).

Metrics	Background	Lifestyle	History	All
**60 Train – 40 Test**				
Accuracy	0.386	0.383	0.744	0.715
Precision	0.236	0.305	0.751	0.707
Recall	0.386	0.383	0.744	0.715
F1 Score	0.264	0.301	0.735	0.700
**50 Train – 50 Test**				
Accuracy	0.371	0.375	0.720	0.705
Precision	0.213	0.361	0.721	0.708
Recall	0.391	0.385	0.720	0.715
F1 Score	0.239	0.326	0.692	0.706

SVM obtained the highest accuracy, with scores of 77%, 80%, 91% and 90% on background, lifestyle, history of grades and all features respectively in 50–50 train–test splits for binary classification (pass or fail). SVM also obtained highest accuracy for the five-class classification (grade A, B, C, D and F) with 39%, 38%, 73% and 71% for the four categories respectively. Based on the results, history of student grades shows significant contribution to a good student performance, where the classification rates obtained are the highest among the four respective categories in each respective classifier. This finding is consistent with the observations from Hwang *et al.,* ^16^ Mega *et al*. ^17^ and Waheed *et al.,* ^18^ that the students’ performance is highly related to the history of grades.

Table 12 shows the comparison of our models with other research work in 50–50 train–test splits for binary classification (pass or fail) on the dataset with population of two Portuguese secondary schools.

**Table 12.** Comparison of our models with others research work on two Portuguese secondary schools.

Model and features	Data		
	Mathematics (395 students)	Portuguese (649 students)	Mathematics and Portuguese (1044 students)
SVM on all features [our model]	-	-	0.90
SVM on history of grades [our model]	-	-	0.91
SVM on all features ^4^	0.89	-	-
Naive predictor on all features ^17^	0.92	0.90	-
SVM on all features ^17^	0.86	0.91	-

## Conclusions

The paper presented predictive modelling of student performance based on four categories. Based on the results, history of student grades shows significant contribution to a good student performance. SVM obtained the highest accuracy with scores of 77%, 80%, 91% and 90% on background, lifestyle, history of grades and all features respectively in 50-50 train-test splits for binary classification (pass or fail). SVM also obtained highest accuracy for five class classification (grade A, B, C, D and F) with 39%, 38%, 73% and 71% for the four categories respectively. The results show that the history of grades form significant influence on the student performance. The study looks at data only from Portugal and may not reflect a general view of the case. Future work will include more datasets from different countries. Also, other classifiers will be explored and investigated.

## Data availability

### Underlying data

Kaggle: A machine learning approach to predictive modelling of student performance

https://www.kaggle.com/larsen0966/student-performance-data-set

and

https://archive.ics.uci.edu/ml/datasets/Student+Performance

Data are available under the terms of the Creative Commons Zero “No rights reserved” data waiver (CC0 1.0 Public domain dedication).

## Ethics approval

Ethical Approval Number: EA1612021 (From Technology Transfer Office (TTO), Multimedia University).
