# Supplementary material for: A Machine Learning Approach to Predictive Modelling of Student Performance
Source: F1000Res. 2022 May 23;10:1144. Originally published 2021 Nov 11. [Version 2] doi: 10.12688/f1000research.73180.2 (PMC9194521; doi:10.12688/f1000research.73180.2)
Supplement: Peer review history

# Peer review: Reviewer response for version 2

**DOI:** 10.5256/f1000research.134172.r138617

**Published:** 2022-06-13

**Authors:** Huiling Chen

**Affiliations:** 1 College of Computer Science and Artificial Intelligence, Wenzhou University, Wenzhou, China

The authors have solved all the comments, now the paper is ready for indexing.

Is the work clearly and accurately presented and does it cite the current literature?

Yes

If applicable, is the statistical analysis and its interpretation appropriate?

Yes

Are all the source data underlying the results available to ensure full reproducibility?

Yes

Is the study design appropriate and is the work technically sound?

Yes

Are the conclusions drawn adequately supported by the results?

Yes

Are sufficient details of methods and analysis provided to allow replication by others?

Yes

Reviewer Expertise:

NA

I confirm that I have read this submission and believe that I have an appropriate level of expertise to confirm that it is of an acceptable scientific standard.

# Peer review: Reviewer response for version 2

**DOI:** 10.5256/f1000research.134172.r138618

**Published:** 2022-05-24

**Authors:** Sadiq Hussain

**Affiliations:** 1 System Administrator, Dibrugarh University, Dibrugarh, Assam, India

All my queries and concerns were handled and the article may be accepted for indexing.

Is the work clearly and accurately presented and does it cite the current literature?

Partly

If applicable, is the statistical analysis and its interpretation appropriate?

Yes

Are all the source data underlying the results available to ensure full reproducibility?

Yes

Is the study design appropriate and is the work technically sound?

Yes

Are the conclusions drawn adequately supported by the results?

Partly

Are sufficient details of methods and analysis provided to allow replication by others?

Yes

Reviewer Expertise:

Educational Data Mining, Medical Analytics

I confirm that I have read this submission and believe that I have an appropriate level of expertise to confirm that it is of an acceptable scientific standard.

# Response

**Published:** 2022-05-24

**Authors:** Hu NG

**Affiliations:** Multimedia University, Malaysia

Dearest Reviewer,

 Thank you for the comments. Thank you for everything.

# Peer review: Reviewer response for version 1

**DOI:** 10.5256/f1000research.76815.r136540

**Published:** 2022-05-12

**Authors:** Huiling Chen

**Affiliations:** 1 College of Computer Science and Artificial Intelligence, Wenzhou University, Wenzhou, China

The authors propose a data mining approach to identify significant factors and predict student performance, based on two datasets collected from two secondary schools in Portugal. The overall structure of the article is also reasonable. The interpretation and description of the experimental results are also detailed. The experimental results show that the proposed method is excellent in terms of accuracy, precision, recall, and F1 score. In my opinion, this method has some practical value.

 However, this manuscript suffers from a number of weak points, it should be further improved before being considered for indexing. Let’s elaborate on some of them:

1. In the results of the Abstract, the authors summarize the classification results of SVM on the dataset. However, we do not see the impact and contribution of the proposed method on the experimental results.
2. Introduction - include the description of the innovations, contributions, and the structure of the article.
3. In the introduction, I also suggest the authors make a comprehensive investigation on the machine learning method such as the works by Wang *et al.* (2022 ^1^) and Wang *et al.* (2022 ^2^), in the literature in the introduction part and give analysis of the existing works to make the whole work more in-depth.
4. The title of the fourth part of the paper, “Methodology”, should be changed to “Methodology and Experimental Results”.
5. The number of students aged 20 and 21 is not given in Figure 2, is it a problem with the data set？
6. With the experimental results in Table 6-11, there are differences in the results obtained by different classifiers. What is the theoretical basis of the paper for the choice of classifier?
7. In the conclusion, the contributions and flaws of the proposed method are not discussed.

Is the work clearly and accurately presented and does it cite the current literature?

Yes

If applicable, is the statistical analysis and its interpretation appropriate?

Yes

Are all the source data underlying the results available to ensure full reproducibility?

Yes

Is the study design appropriate and is the work technically sound?

Yes

Are the conclusions drawn adequately supported by the results?

Yes

Are sufficient details of methods and analysis provided to allow replication by others?

Yes

Reviewer Expertise:

data mining

I confirm that I have read this submission and believe that I have an appropriate level of expertise to confirm that it is of an acceptable scientific standard, however I have significant reservations, as outlined above.

## References

[1] : Tool for Predicting College Student Career Decisions: An Enhanced Support Vector Machine Framework. *Applied Sciences* .2022;12(9) : 10.3390/app12094776 10.3390/app12094776

[2] : Lupus nephritis diagnosis using enhanced moth flame algorithm with support vector machines. *Comput Biol Med* .2022;145: 10.1016/j.compbiomed.2022.105435 105435 10.1016/j.compbiomed.2022.105435 35397339

# Response

**Published:** 2022-05-17

**Authors:** Hu NG

**Affiliations:** Multimedia University, Malaysia

Dear Prof Huiling Chen,

 We are greatly appreciative of the insightful comments and helpful suggestions that you have provided.

 The following are our response on the issues that you have highlighted:

 Your comment:

1. In the results of the Abstract, the authors summarize the classification results of SVM on the dataset. However, we do not see the impact and contribution of the proposed method on the experimental results.

**Our response:**

 Thank you for the comments. From this research work, we found out that history of grades forms significant influence on the student performance. This is the main impact and contribution.

 Your comment:

 2. Introduction - include the description of the innovations, contributions, and the structure of the article.

**Our response:**

 The last 2 paragraphs of the Introduction have been rewritten to reflect this.

 The contributions of this paper are the identification of significant features that influence student assessment, which in turn can be used to develop various predictive models to ascertain student performance. This will assist educators to form corrective or remedial actions can help to improve student performance. In addition, this may also assist in formulating curriculums that may direct students to career pathways that are most suitable for them.

 The paper is structured as follows: Related Works, Methodology and Results, followed by Conclusions.

 Your comment:

 3.  In the introduction, I also suggest the authors make a comprehensive investigation on the machine learning method such as the works by Wang *et al.* (2022 ^1^ ) and Wang *et al.* (2022 ^2^ ), in the literature in the introduction part and give analysis of the existing works to make the whole work more in-depth.

**Our response:**

 We have added one of the papers recommended into the Related Works. Thank you for the recommendation.

 Wang, Z., Liang, G., & Chen, H. (2022). Tool for Predicting College Student Career Decisions: An Enhanced Support Vector Machine Framework. Applied Sciences, 12(9), 4776.

 Your comment:

 4.  The title of the fourth part of the paper, “Methodology”, should be changed to “Methodology and Experimental Results”.

**Our response:**

 The title has been rephrased to ‘Methodology and Results’.

 Your comment:

 5.  The number of students aged 20 and 21 is not given in Figure 2, is it a problem with the data set？

**Our response:**

 Figure 2 had been edited to show the number of students aged 20 and aged 21.

 Your comment:

 6.  With the experimental results in Table 6-11, there are differences in the results obtained by different classifiers. What is the theoretical basis of the paper for the choice of classifier?

**Our response:**

 Due to previous work, we found out that these classifiers work well for our use cases, that is why in this work we have only applied these. We will compare other classifiers in future work.

 Your comment:

 7.  In the conclusion, the contributions and flaws of the proposed method are not discussed.

**Our response:**

 The conclusions have been revised to include the significant results, contribution and the weakness, which will be addressed in future work.

# Peer review: Reviewer response for version 1

**DOI:** 10.5256/f1000research.76815.r99883

**Published:** 2021-11-18

**Authors:** Sadiq Hussain

**Affiliations:** 1 System Administrator, Dibrugarh University, Dibrugarh, Assam, India

The paper presented a predictive model on student performance based on the data from schools in Portugal. Student grades are the most important feature observed in the study. The study is complete from an experimental perspective, but it needs improvement in related works and the introduction section. After these modifications, the study will be approvable.

1. There are grammatical errors in this paper, which should be revised. 
2. The literature review should be more in detail and add at least five more papers.
3. The conclusion should also be elaborated a little more. The major findings in their study should be discussed. For example, out of the classifiers applied, which classifier demonstrated the best accuracy? An evaluation of the methodology that the authors deployed would be welcome. 
4. The introduction section should also focus on the research problem. Why this kind of research is beneficial, and to whom? How can management take advantage of it and how can companies evaluate these results to find the best students/universities for job placements?
5. Was the dataset balanced? What was the imbalance ratio?
6. Kindly provide justification for using the applied classifiers only: why were other classifiers not considered?
7. What were the most influential features in student performance? Was there any unnecessary feature that was taken into account?

Is the work clearly and accurately presented and does it cite the current literature?

Partly

If applicable, is the statistical analysis and its interpretation appropriate?

Yes

Are all the source data underlying the results available to ensure full reproducibility?

Yes

Is the study design appropriate and is the work technically sound?

Yes

Are the conclusions drawn adequately supported by the results?

Partly

Are sufficient details of methods and analysis provided to allow replication by others?

Yes

Reviewer Expertise:

Educational Data Mining, Medical Analytics

I confirm that I have read this submission and believe that I have an appropriate level of expertise to confirm that it is of an acceptable scientific standard, however I have significant reservations, as outlined above.

# Response

**Published:** 2022-05-17

**Authors:** Hu NG

**Affiliations:** Multimedia University, Malaysia

Dear Prof Sadiq Hussain,

 We are greatly appreciative of the insightful comments and helpful suggestions that you have provided.

 The following are our response on the issues that you have highlighted:

 Your comment:

 a) There are grammatical errors in this paper, which should be revised.

**Our response:**

 The paper has been proofread for grammatical errors. Thank you for pointing this out.

 Your comment:

 b) The literature review should be more in detail and add at least five more papers.

**Our response:**

 Six papers on student dropout, interpersonal influences, as well as career decisions have been added to the Literature Review.

  

1. Hussain, S., Dahan, N. A., Ba-Alwib, F. M., & Ribata, N. (2018). Educational data mining and analysis of students’ academic performance using WEKA. Indonesian Journal of Electrical Engineering and Computer Science, 9(2), 447-459.
2. Chung, J.Y.; Lee, S. Dropout early warning systems for high school students using machine learning. Child. Youth Serv. Rev. 2019, 96, 346–353.
3. Nauta, M.M.; Saucier, A.M.; Woodard, L.E. Interpersonal influences on students’ academic and career decisions: The impact of sexual orientation. Career Dev. Q. 2001, 49, 352–362.
4. Lee, P.C.; Lee, M.J.; Dopson, L.R. Who influences college students’ career choices? An empirical study of hospitality management students. J. Hosp. Tour. Educ. 2019, 31, 74–86.
5. Kim, S.-Y.; Ahn, T.; Fouad, N. Family influence on Korean students’ career decisions: A social cognitive perspective. J. Career Assess. 2016, 24, 513–526.
6. Wang, Z., Liang, G., & Chen, H. (2022). Tool for Predicting College Student Career Decisions: An Enhanced Support Vector Machine Framework. Applied Sciences, 12(9), 4776.

Your comment:

 c) The conclusion should also be elaborated a little more. The major findings in their study should be discussed. For example, out of the classifiers applied, which classifier demonstrated the best accuracy? An evaluation of the methodology that the authors deployed would be welcome.

**Our response:**

 The conclusion has been elaborated with the results. The following has been added:

 SVM obtained the highest accuracy with scores of 77%, 80%, 91% and 90% on background, lifestyle, history of grades and all features respectively in 50-50 train-test splits for binary classification (pass or fail). SVM also obtained highest accuracy for five class classification (grade A, B, C, D and F) with 39%, 38%, 73% and 71% for the four categories respectively.

 Your comment:

 d) The introduction section should also focus on the research problem. Why this kind of research is beneficial, and to whom? How can management take advantage of it and how can companies evaluate these results to find the best students/universities for job placements?

**Our response:**

 The following statements have been added to the Introduction to address this:

 This will assist educators to form corrective or remedial actions can help to improve student performance. In addition, this may also assist in formulating curriculums that may direct students to career pathways that are most suitable for them.

 For management and companies, this is not in the scope of this research and will be explored in future work.

 Your comment:

 e) Was the dataset balanced? What was the imbalance ratio?

**Our response:**

 The original datasets are not balanced due to the distribution of grades. In order to overcome this, in this work we performed discretization, to form 2 groups – binary levels and 5 levels. As per referred to the work from

  

1. Hussain, S., Dahan, N. A., Ba-Alwib, F. M., & Ribata, N. (2018). Educational data mining and analysis of students’ academic performance using WEKA. Indonesian Journal of Electrical Engineering and Computer Science, 9(2), 447-459.

Your comment:

 f) Kindly provide justification for using the applied classifiers only: why were other classifiers not considered?

**Our response:**

 Due to previous work, we found out that these classifiers work well for our use cases, that is why in this work we have only applied these. We will compare other classifiers in future work.

 Your comment:

 g) What were the most influential features in student performance? Was there any unnecessary feature that was taken into account?

**Our response:**

 The most influential features come from the history of student grades (Table 3). The following statement can be found in the discussions of the classification result:

 Based on the results, history of student grades shows significant contribution to a good student performance, where the classification rates obtained are the highest among the four respective categories in each respective classifier.

 No unnecessary features were taken into account.
